# Isotope Effects as Probes for Enzyme Catalyzed Hydrogen-Transfer Reactions

**DOI:** 10.3390/molecules18055543

**Published:** 2013-05-14

**Authors:** Daniel Roston, Zahidul Islam, Amnon Kohen

**Affiliations:** Department of Chemistry, The University of Iowa, Iowa City, IA 52242, USA

**Keywords:** kinetic isotope effects, Marcus-like models, alcohol dehydrogenase, thymidylate synthase, hydrogen tunneling

## Abstract

Kinetic Isotope effects (KIEs) have long served as a probe for the mechanisms of both enzymatic and solution reactions. Here, we discuss various models for the physical sources of KIEs, how experimentalists can use those models to interpret their data, and how the focus of traditional models has grown to a model that includes motion of the enzyme and quantum mechanical nuclear tunneling. We then present two case studies of enzymes, thymidylate synthase and alcohol dehydrogenase, and discuss how KIEs have shed light on the C-H bond cleavages those enzymes catalyze. We will show how the combination of both experimental and computational studies has changed our notion of how these enzymes exert their catalytic powers.

## 1. Introduction

One of the most powerful tools in the chemist’s arsenal for studying reaction mechanisms and transition state structures is to measure kinetic isotope effects (KIEs). KIEs have become a pivotal source of information on enzymatic reactions, and a number of recent reviews have described the successes of KIEs in enzymology, from determining molecular mechanisms and transition state structures that are useful for drug design [1], to answering questions on the roles of nuclear quantum tunneling [2,3], electrostatics [4], and dynamic motions [5,6,7,8] in enzyme catalyzed reactions.

Despite these many achievements, the enzymology community is still in need of a comprehensive model of catalysis. A current intense debate, for example, over the possible role of non-statistical dynamics in enzyme catalysis [9,10,11,12,13,14,15,16,17], shows little sign of abating. The present review seeks to provide an updated description of the achievements of KIEs in progressing toward a more comprehensive theory, as well as to highlight the remaining questions that KIEs may be suited to answer in the future. We will begin with a thorough theoretical background on the physical sources of KIEs and general schemes for measuring and interpreting them in the context of the complex kinetic cascades of enzymatic reactions. We will then proceed to describe how KIEs have illuminated two model reactions: alcohol dehydrogenase (ADH) and thymidylate synthase (TSase). These two case studies provide detailed examples of the many practical uses of KIEs for answering questions on the nature of enzymatic hydrogen transfers.

## 2. Theory of KIEs

### 2.1. Semi-Classical Models of KIEs

Models that ignore nuclear quantum effects typically (although not always, see [18]) predict no isotope effects, because the isotopes do not affect the electronic potential surface for the reaction under study. Semi-classical models refer to those models that ignore contributions of quantum mechanical tunneling, *i.e.*, only consider quantum mechanical effects on vibrational zero point energy. We will discuss tunneling in detail later, but for historical perspective and because of semi-classical models’ usefulness to heavy atom KIEs, we will describe those models first. Such models are rooted in transition state theory (TST), which assumes a dynamic equilibrium between the transition state (TS) and the ground state (GS). The rate constant (*k*) described by TST is:


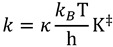
(1)

Here 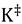 is the equilibrium constant between the TS and GS, 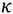 is the transmission coefficient (which can be smaller than unity due to friction [19] or recrossing [20], or be larger than unity due to tunneling as discussed below), T is the absolute temperature, h is Planck’s constant and *k*_B_ is Boltzmann’s constant. The equilibrium constant, 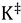 can be expressed in terms of partition functions as follows:


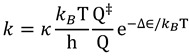
(2)

Here 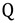 and 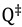 are the total partition functions of the GS and TS respectively and 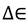 is the energy difference between the TS and GS. A KIE is the ratio of rate constants for a reaction involving a light isotope (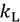) and a heavy isotope (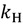). Taking this ratio using the TST rate constants of Equation (2) yields the Bigeleisen equation [21,22]:


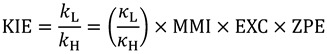
(3)

Here 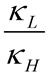 is the ratio of transmission coefficients, which semi-classically (no tunneling) is close to unity. MMI, the “Mass Moment of Inertia” term, refers to the isotope effect on translation and rotation. In the vast majority of reactions, isotope effects on translation and rotation are very small because isotopic substitution does not significantly perturb the system’s overall mass or moment of inertia, so the MMI term is usually smaller than one but close to unity [23]. EXC refers to the isotopic variations on excited vibrational levels. This term is bit larger than one but close to unity, since excited vibrational states have very small populations even at relatively high temperatures. The product of these two terms (MMI and EXC) is very close to unity and is usually negligible for hydrogen KIEs, but may make a more significant contribution to heavy atom KIEs because the relative contributions from other effects is smaller. ZPE is the contribution arising from the isotopic difference in vibrational zero-point energies, and is the primary contributor to KIEs in semi-classical models. Heavier isotopes have lower vibrational ZPEs at both the GS and TS, requiring different amounts of thermal activation to reach the TS. Thus, Equation (3) can be approximated as follows: 


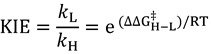
(4)

Here 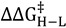 is the difference in the free energy of activation between isotopologues.

### 2.2. Primary Kinetic Isotope Effects

**Figure 1:**
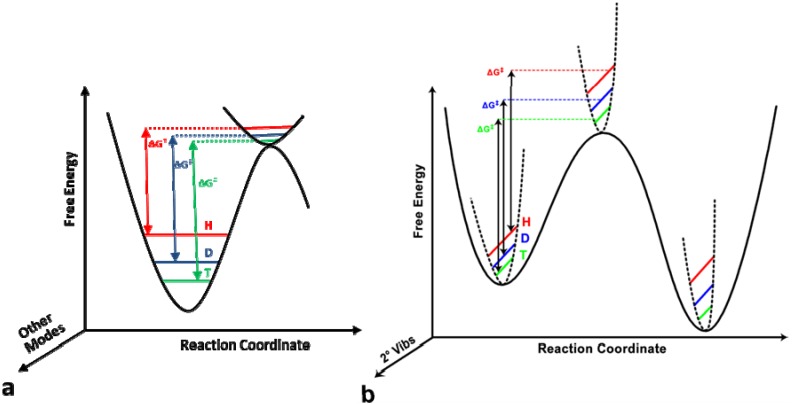
(**a**) Semi-classical model of 1° KIEs. KIEs arise from the difference in ZPE between the reactants at the GS and the TS. In this schematic, the reaction coordinate is a C-H stretch that becomes a translation in the TS, so the ZPE of that mode is lost. Isotopically sensitive vibrations orthogonal to the reaction coordinate are generally present at the TS, as well as the GS (although orthogonal GS modes are omitted here for clarity). (**b**) Semi-classical model of 2° KIEs for a reaction where the isotopically sensitive vibrational mode increases in frequency at the TS, yielding an inverse 2° KIE, and the ZPE in this case increases even more in the product, yielding an inverse equilibrium isotope effect (EIE). Figure 1b reproduced with permission from ref. [8].

A primary (1°) KIE is a KIE where the isotopically labeled atom is directly involved in bond cleavage or formation. For most organic and biological reactions, the reaction coordinate can be thought of as a stretching vibrational mode at the GS becoming a translational mode at the TS. Thus, the GS-ZPE of the stretching mode is lost and only the much lower frequencies of vibrations orthogonal to the reaction coordinate are present at the TS (Figure 1), so a first-order approximation of the source of KIEs is just the difference in GS-ZPE between the two isotopes:


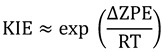
(5)

This approximation provides a semi-classical upper limit on KIEs, which, for C-H (GS frequency of 3000 cm^−1^) cleavage at 25 °C is 6.9 and 15.8 for 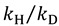
and 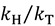 , respectively [23].

However, the vibrational frequencies orthogonal to the reaction coordinate can deflate the primary KIEs from the maximum predicted values (Figure 1). Smaller KIEs can also be observed if the primary motion along the reaction coordinate is a bending mode, which has lower GS-ZPE, if recrossing is significant, in cases of exothermic or endothermic reactions. A typical use of 1° KIEs is to determine the symmetry and structure of the TS. The above approximation is most valid for a linear symmetric and dissociated TS. Thus, the size of the primary KIE reaches a maximum in that case. An associated TS, or reactant-like (very early, for exothermic reaction) or product-like (very late, for endothermic reaction) TS reduces the primary KIE from the semi-classical upper limit as the assumption of completely losing ZPE in the TS is less valid in these cases [24].

### 2.3. Secondary KIEs

**Figure 2:**
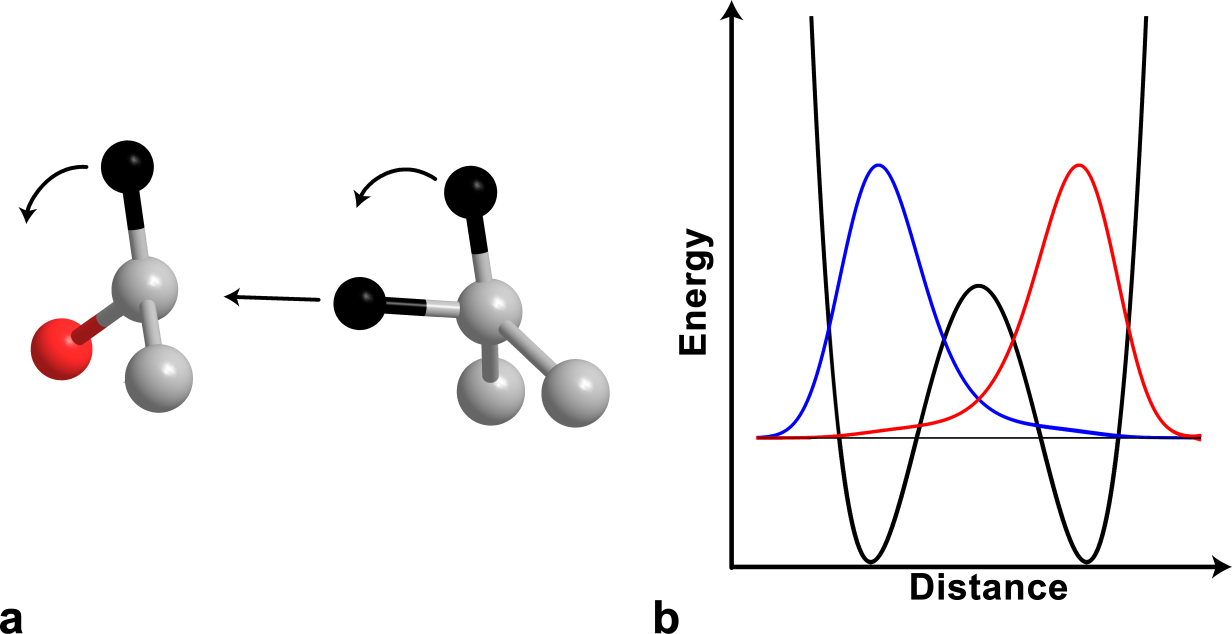
The model of tunneling and coupled motion. (**a**) The reaction coordinate involves motion of both the 1° and 2° atoms, which tunnel through the reaction barrier. (**b**) The efficiency of tunneling is proportional to the overlap between donor (blue) and acceptor (red) wave functions. Reproduced with permission from ref. [8].

Secondary (2°) KIEs refer to KIEs resulting from isotopic substitution of an atom not directly involved in bond cleavage or formation. These KIEs result from changes in the vibrational frequencies of modes orthogonal to the reaction coordinate (Figure 2b). In this situation; the ZPE of the isotopically labeled atom is not completely lost at the TS; in fact; in many cases the ZPE of that atom *increases*. The magnitude of 2**°** KIEs; therefore; is generally smaller than primary KIEs. 2° KIEs are referred to as “normal” if the lighter isotopes react faster than heavier isotopes 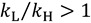; and “inverse” when heavier isotopes react faster than lighter isotopes 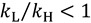. An inverse KIE indicates that the ZPE of the isotopically sensitive modes increases in going from reactants to TS.

2° KIEs can be further divided into α- and β-2**°** KIEs, depending on whether isotopic substitution is made at the α- or β-position relative to the bond being cleaved or formed. α-2° KIEs (isotopic substitution of an atom directly bonded to an atom involved in bond cleavage/formation) typically report on the extent to which the central atom changed its hybridization in going from GS to TS. β-2° KIEs, on the other hand, refer to isotopic substitution at a position two bonds away from a reacting atom, and report on the extent of charge formation at the TS, since different isotopes allow for differing levels of hyper conjugation [25]. In semi-classical theory, the direction (normal or inverse) and magnitude of 2° KIEs for many types of reactions are related to equilibrium isotope effects (EIE). The EIE is the isotope effect on an equilibrium constant:


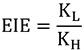
(6)

Here K_L_ and K_H_ are the equilibrium constants for the light and heavy reactions, respectively. In contrast to 2**°** KIEs, the magnitude and direction of EIEs depend on the ZPE of the isotopically labeled vibrations at the reactant and product ground states. If the ZPE changes monotonically (*i.e.*, either increasing or decreasing throughout the reaction), then the 2° KIE will fall between the EIE and unity. For example, if the hybridization of the central carbon changes from sp^3^ in the reactant to sp^2^ in the product, the α-**2**° EIE for a hydrogen bonded to that carbon will be greater than unity as the H gains bending freedom in going from sp^3^ to sp^2^. Thus, the difference in ZPE (ΔZPE) between reactant and product favor depletion of the heavy isotope in product. Comparisons of 2° KIEs with EIEs have traditionally been used to determine the position of the TS. 2° KIEs very close to unity indicate a very early TS, whereas a very late TS results in 2° KIEs close to the EIE. However, there are a few reports of cases where 2° KIEs fell outside of the aforementioned range (unity to EIE) and this has indicated non-classical behavior like tunneling (see below on ADH) [26,27,28,29]. Note that for certain types of reactions, the ZPE does not change monotonically. For example, in an S_N_2 reaction, the central carbon is penta-coordinate at the TS, so α-hydrogens have very little vibrational freedom and the ZPE is thus higher at the TS than in either the reactants or products. In this case, the EIE is likely to be close to unity, so a small KIE can indicate either an early or a late TS, whereas a large KIE indicates a symmetric TS.

### 2.4. Intrinsic KIEs

Enzymatic reactions involve multiple kinetic steps in addition to the bond cleavage/formation step (referred to as the “chemical step”), including substrate binding, conformational changes and product release, with product release often being the rate-limiting step. In cases where these steps are isotopically insensitive, they may still mask the isotope effect on the chemical step, making the observed KIEs appear smaller than the intrinsic KIEs (KIE_int_, the KIE on the chemical step of interest), and this phenomenon is known as “kinetic complexity”. 

As an illustration of the effects of kinetic complexity, consider the following reaction mechanism:


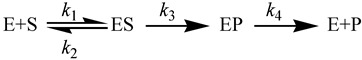
(7)

Here conversion of the enzyme-substrate complex (ES) to the enzyme-product complex (EP) is the chemical step. In such a mechanism, the observed KIE (KIE_obs_) will be deflated from KIE_int_ by an amount depending on the “commitments to catalysis”. The forward commitment (C_f_) is defined as the ratio of the isotopically sensitive rate constant (*k*_3_ in the simple mechanism of Equation (7)) to the net rate constant for the breakdown of the reactive ES complex to E and S (in this case, just *k*_2_). Thus, for the above reaction mechanism, C_f_ = *k_3_/k_2_*. The reverse commitment (C_r_) is the ratio of the rate of the isotopically sensitive step in the reverse direction (from EP to ES) to the isotopically insensitive net rate constant that decomposes the EP complex to E and P. In the above reaction mechanism (Equation (7)), the reverse commitment is zero, as the isotopically sensitive step is irreversible. KIE_obs_ on the second order rate constant (*V/K*, *i.e.*, *k*_cat_/*K*_M_),is affected by commitments as follows [30]:


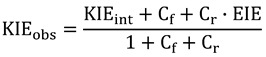
(8)

In cases like the above mechanism, where C_r_ = 0, Equation (8) simplifies to:


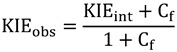
(9)

Observed KIEs will be the same as intrinsic KIEs only if both C_r_ and C_f_ are negligible. Otherwise, there are several ways to better expose the chemical step and assess intrinsic KIEs, such as the Northrop method [31] (see below), pre-steady-state measurements, using alternative substrates, mutagenesis, changing pH or changing temperature. The Northrop method [31] assesses intrinsic KIEs from the observed KIEs by exploiting the Swain-Schaad relationship [32]. In the following section we will discuss the Swain-Schaad relationship and its application in the Northrop method. 

### 2.5. Swain-Schaad Relationships

The Swain-Schaad relationship is the proportionality between different hydrogen KIEs (e.g., between *k*_H_/*k*_D_ and *k*_H_/*k*_T_ or *k*_D_/*k*_T_). A relatively simple derivation [32] based on the assumptions of the Bigeleisen equation and harmonic vibrational modes, shows that hydrogen KIEs are related to one another by simple exponential relationships:


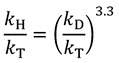
(10)


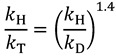
(11)


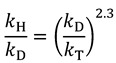
(12)

where the exponent only depends on the reduced masses of the different isotopes, which for Equation (11), for example, is:


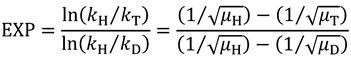
(13)

where *μ*_i_ is the reduced mass of the C_i_ bond under study (where i is H, D, or T). In these equations, the exponent is referred to as the Swain-Schaad exponent (SSE). Using the SSE, Northrop developed a method to extract intrinsic KIEs by measuring multiple KIEs. In a situation where Cr is zero, Equation (9) can be rewritten as follows:


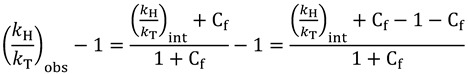
(14)


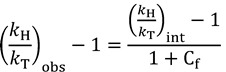
(15)

Or, for H/D KIEs, one obtains:


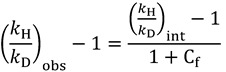
(16)

Dividing Equation (16) by Equation (15), we are left with the following expression:


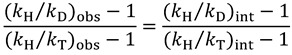
(17)

Application of the Swain-Schaad relationship via Equation (11) then gives:


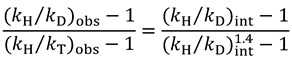
(18)

Thus, by measuring both 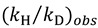 and 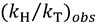, one can assess 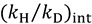. A recent study showed the usefulness of all three combinations of hydrogen isotopes in assessing intrinsic KIEs for an enzyme affected by kinetic complexity [33]. The limitations and assumption associated with this method are discussed in detail in ref. [33] and many cited therein.

### 2.6. Breakdown of the Semi-Classical Model: Quantum Tunneling

Since the 70s, a variety of experiments have shown that not all data can be explained by semi-classical models, particularly in reactions involving the transfer of hydrogen (H^+^, H^−^, or H∙). One of the first indications of a breakdown of the semi-classical model was the discovery by Cleland and co-workers that 2° KIEs in ADH [26] and formate dehydrogenase [29] fell outside of the semi-classical limits of the EIE to unity. The researchers proposed quantum mechanical tunneling and 1°–2° coupled motion (Figure 2). Tunneling is the phenomenon where a particle’s probability density spreads through a potential energy barrier without ever obtaining the energy necessary to surmount that barrier. Since a particle’s ability to tunnel depends on mass, KIEs are an excellent probe for tunneling effects. Further evidence for tunneling came from the breakdown of the Swain-Schaad relationship in so-called “mixed labeling” experiments, which measure the 2° H/T KIE with H at the 1° position, and the 2° D/T KIE with D at the 1° position [34,35,36]. Another experimental finding that could not be explained semi-classically are 1° KIEs with magnitudes drastically inflated from the semi-classical limits [37] or with temperature dependence outside the semiclassical predicted range [7,8,23,36,37,38,39].

In the semi-classical model, KIEs originate from the difference in activation energy of the isotopologues (ΔE_a_) and the KIEs can be modeled with the Arrhenius equation, which for KIEs is identical to the Eyring equation:


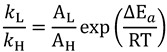
(19)

where A_L_/A_H_ is the isotope effect on the Arrhenius pre-exponential factor, which is expected to be close to unity (e.g., 0.5 < A_H_/A_D_ < 1.47) [40]. In some cases, the observed isotope effect on the pre-exponential factor is greater than unity, corresponding to smaller than expected temperature dependence of KIEs, whereas other reactions exhibit an isotope effect on the pre-exponential factor that is smaller than unity, corresponding to larger than expected temperature dependence. Several attempts to account for these deviations from semi-classical behavior have arisen over the years.

### 2.7. Models of Tunneling

**Figure 3:**
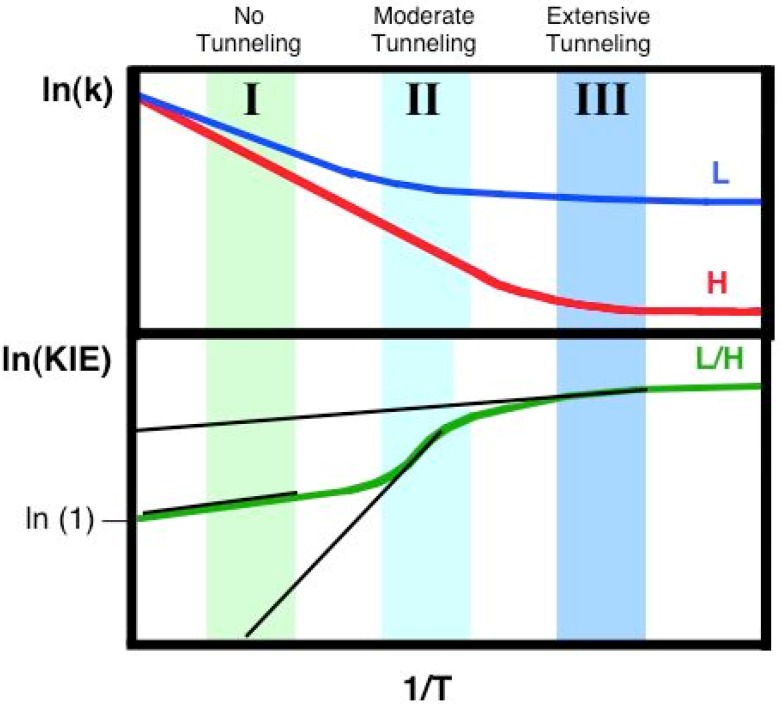
Arrhenius plot of rates (top panel) and KIEs (bottom panel, green line) of light (L, in blue) and heavy (H, in red) isotopes. In region I tunneling is negligible so A_L_/A_H_ isclose to unity. Region II is the moderate tunneling region where only the lighter isotope tunnels and the correspondingA_L_/A_H_ is much smaller than unity. Both isotopes tunnelin region III, making the A_L_/A_H_ much higher than unity.Reproduced with permission from ref. [42].

Some formalisms add a tunneling correction term to the TST rate constant, giving rate constants of the form:


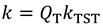
(20)

where *k*_TST_ is the TST rate constant and Q_T_ is a correction for tunneling effects. For example, the Bell correction [41] used a parabolic reaction barrier and was able to account for some rate and KIE behavior as illustrated in Figure 3. In the high temperature limit (region I in Figure 3), reactions mostly take place by thermal activation which leads to temperature-dependent rates and KIEs, reflecting the differences in activation energy between two isotopologues (light in blue and heavy in red), and the corresponding A_L_/A_H_ is close to unity, as predicted by semi-classical models [40]. In the low temperature limit, very little thermal energy is available for activation; therefore, reactions take place exclusively by tunneling, leading to temperature independent rates and KIEs (Figure 3, region III) and A_L_/A_H_ much higher than unity. Between these two extremes lies the moderate tunneling region II where the lighter isotope mostly tunnels, but the heavier isotope reacts mostly by thermal activation. This results in a very steep temperature dependence of KIEs and A_L_/A_H_ much smaller than unity due to a temperature dependent rate for the heavy isotope, but temperature independent rate for the light isotope.

Such tunneling correction models can reproduce temperature-dependent or temperature independent KIEs [43], but cannot yield temperature-dependent rates with temperature-independent KIEs, which is precisely what numerous experiments have reported for various systems [7,8,36,39,42,44]. We note that several high level simulations [45,46,47,48,49] have used sophisticated tunneling corrections to TST, along with other corrections, to reproduce the temperature dependence of experimental rates and KIEs, but such corrections are outside the scope of this review.

**Figure 4:**
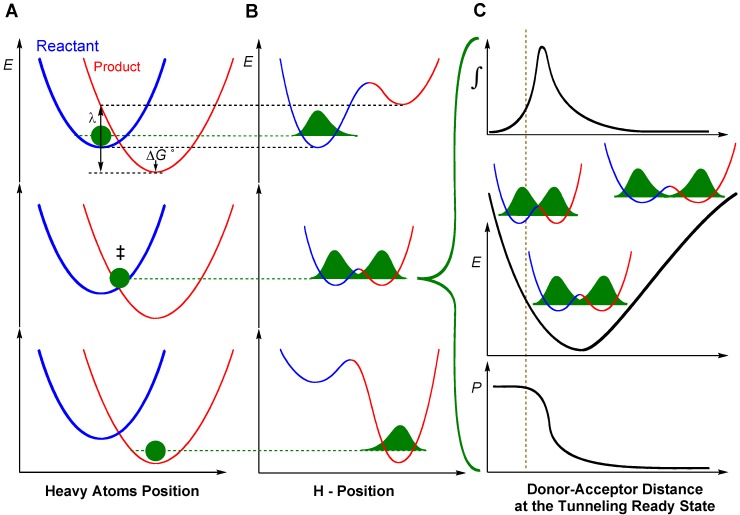
Marcus-Like models of hydrogen tunneling—a visualization of Equation (21). Three slices of the potential energy surface (PES) along components of the collective reaction coordinate showing the effect of heavy-atom motions on the zero point energy in the reactant (blue) and product (red) potential well. Panel **A** presents the heavy atom coordinate (also known as the Marcus parabolas), and Panel **B** shows the H-atom position, which is orthogonal to the heavy atom coordinate. In the top panels the hydrogen is localized in the reactant well, and the zero point energy of the product state is higher than that of the reactant state. Heavy atom reorganization brings the system to the tunneling ready state (TRS, middle panels **A** and **B**), where the zero point energy in the reactant and product wells are degenerate and the hydrogen can tunnel between the wells. Further heavy atom reorganization breaks the transient degeneracy and traps the hydrogen in the product state (bottom panels). The rate of reaching the TRS depends on the reorganization energy (λ) and driving force (ΔG°), which are indicated in the top panel, and further discussed under Equation (21). Panel **C** shows the effect of DAD sampling on the wave function overlap at the TRS (middle panel). Transmission probability (*P*) is a function of the overlap integral of the hydrogen wave functions in the reactant (blue) and product (red) wells (bottom panel **C**). The top panel **C** presents the contribution to H-transfer at each DAD as a function of the *P* and the population at each DAD (*i.e.*, the integrated terms in Equation (21)). The vertical dashed line represents the DAD under which the ZPE is greater than the barrier height. At such distances, the process of a wave function spreading from reactant well to product well is no longer “tunneling”, but one can still use the particle’s transmission probability analogously to the tunneling probability at longer DADs.

To rationalize findings that could not fit the above models, researchers have adapted Marcus Theory of electron tunneling [50] to the case of hydrogen tunneling. Full tunneling models of this type are referred to as Marcus-like models (Figure 4), but have also been called by a variety of other names such as environmentally coupled tunneling, vibrationally enhanced tunneling, tunneling promoting vibrations and others [5,6,7,8,9,39,51,52,53,9,39,51]. The underlying feature of Marcus-like models is the separation of light atom motion (*i.e.*, hydrogen tunneling) from heavy atom motion, which includes both the protein environment as well as the surrounding solvent. According to these models, rates are governed by three major processes: 1) heavy atom motion that brings the system to a tunneling ready state (TRS), where the reactant and product potential surfaces are degenerate and tunneling can occur, 2) fluctuations of the tunneling barrier, modulated by heavy atom motion, and 3) the actual process of hydrogen tunneling through the barrier. A general functional form for the rate constant in such models is [5,51,54,55,56]:


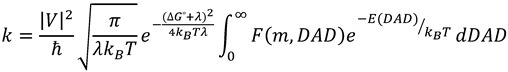
(21)

In this equation, the factors in front of the integral give the rate of reaching a TRS based on the electronic coupling between reactant and product (V, the degree of adiabaticity of the reaction), the reorganization energy (λ), and the driving force of the reaction (∆G°). The integral measures the probability of hydrogen transfer once the system reaches a TRS. The first factor inside the integral determines the probability of tunneling as a function of mass (m) and the donor-acceptor distance (DAD). The last integrated exponential is a Boltzmann factor, giving the probability of being at any given DAD. 

Since the rate of reaching a TRS (the factors outside the integral) is governed by heavy atom motion, it essentially isotopically insensitive, though it governs some of the temperature dependence of the reaction rate. The Boltzmann and tunneling factors, however, make the integral isotopically sensitive and potentially sensitive to temperature, depending on the free energy as a function of DAD, E(DAD) in Equation (21). Thus, Marcus-like models can explain temperature-dependent or temperature-independent rates with temperature-dependent or temperature-independent KIEs. Please note that for DADs shorter than the vertical line in panel C of figure 4, the ZPE is above the barrier, thus the reaction is practically over-the-barrier.

Interpreting KIEs with this kind of model suggests that the temperature dependence of KIEs is a function of the temperature dependence of the distribution of DADs. That is, temperature independent KIEs result from a very narrow distribution of DADs at the TRS that does not change with temperature. Temperature dependent KIEs, on the other hand, result from a loose active site where the TRS can attain a wide range of DADs at thermal equilibrium, and the distribution of DADs is thus temperature sensitive. In fact, we recently proposed a numerical model [54] suggesting that the systems with the most steeply temperature dependent KIEs may actually result from multiple distinct populations in equilibrium with one another along the DAD coordinate. We will further discuss the utility of interpreting KIEs with Marcus-like models in the following case studies on the enzymes ADH and TSase.

## 3. Alcohol Dehydrogenase

**Scheme 1:**
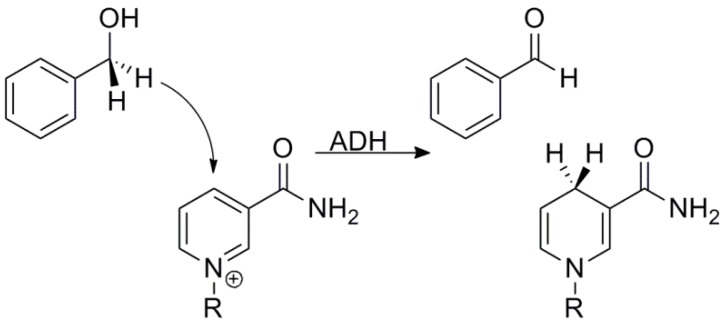
The reaction catalyzed by ADH (R = adenine diphosphate ribosyl-) with BnOH as alternative substrate.

Alcohol dehydrogenase (ADH) has served as one of the classic models for enzymology and specifically, the many uses of isotope effects to study the physical mechanism of hydride transfers [8,57,58,59]. ADH catalyzes the oxidation of an alcohol to an aldehyde using a nicotinamide cofactor (Scheme 1). Some of the benefits of using ADH as a model are that one can study both the forward (alcohol to aldehyde) and reverse reactions using relatively similar conditions, and that for a number of ADHs (e.g., from either yeast or from *Bacillus stearothermophilus*) the hydride transfer step is fully exposed (*i.e.*, kinetic complexity does not interfere with experimental results). Pioneering studies on the hydride transfer in yeast ADH (yADH) by Klinman and coworkers compared isotope effects with linear free energy relationships (Hammett substituent effects) using benzyl alcohols and aldehydes [60,61,62]. While the 1° KIEs were not particularly noteworthy, the 2° KIEs and the substituent effects showed a fascinating contradiction. The α-2° KIEs on the oxidation of benzyl alcohol were close to the EIE (1.35), while those on the reduction of benzaldehyde were close to unity, clearly suggesting an aldehyde-like TS [62]. The substituent effects, however, told the complete opposite story. By measuring rates using benzyl alcohols and benzaldehydes with *para*-substituents possessing a range of electronic effects, Klinman examined the electronic structure of the TS and found that it was more alcohol-like than aldehyde like [60,61]. This blatant contradiction between KIEs and linear free energy relationships was, in retrospect, the first indication that something non-classical was occurring in the reaction, but the idea of tunneling had yet to enter the consciousness of the enzymology community.

Shortly after the publication of those studies, Cleland and coworkers [26,63] measured α-2° KIEs on the nicotinamide cofactor in ADH and found that they were significantly inflated. In fact, despite the fact that the relevant EIE was inverse, as expected for a change from sp^2^ to sp^3^ (reduction of NAD^+^ to NADH), the KIE was significantly greater than unity. The authors interpreted this surprising result as an indication of 1°–2°coupled motion (Figure 3). A theoretical model [64] solidified that interpretation, with the additional component of quantum tunneling of the coupled atoms. The theoretical calculations further suggested that a breakdown of the “rule of the geometric mean” (RGM) could be a strong indicator of tunneling and coupled motion. The RGM is one of the results of the assumptions of the Bigeleisen model of KIEs and states that isotope effects should be independent of one another—there are no isotope effects on isotope effects [65]. Quickly after the predictions of the theoretical model appeared, Cleland and coworkers tested the RGM in the enzyme formate dehydrogenase (FDH), finding that it did not hold, thus supplying very solid evidence for the phenomenon of tunneling and 1°–2° coupled motion [29].

Additional calculations soon provided another important prediction of the model of tunneling and coupled motion. If a reaction involved tunneling and coupled motion, the study asserted, then observed SSEs should deviate significantly from the value predicted by semi-classical theory [66]. Klinman’s group ended up testing this prediction in yADH using what has come to be known as a mixed labeling experiment [34]. In this kind of experiment, the 2° H/T KIE is measured with H at the 1° position, while the 2° D/T KIE is measured with D at the 1° position. These experiments provided a mixed-labeling SSE (mSSE):


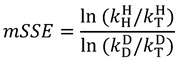
(22)

where 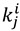 indicates the rate with isotope i at the 1° position and isotope j at the 2° position. Semi-classical models typically predict that mSSE = SSE = 3.3, although some calculations have suggested that even without tunneling the SSE can be somewhat larger than that [27,67]. No semi-classical models, however, can explain the experimental value in yADH, which was over 10, clearly supporting the theory of tunneling and coupled motion [34].

After confirming that inflated mSSEs—and thus tunneling and coupled motion—also occur in horse liver ADH (hlADH) when mutation causes the hydride transfer to be rate-limiting [35], Klinman’s group began to explore the possible role of enzyme structure and dynamics in modulating the tunneling process. A study of a series of active site mutants of hlADH found that based on the measured mSSE for each mutant, the degree of tunneling depends on the hydrogen transfer distance apparent in crystal structures of the mutants [68]. This result suggested that enzymes may have evolved to hold the DAD to a short enough distance for hydrogen tunneling.

Additional information on the mechanisms by which enzymes modulate tunneling came from a study of the temperature dependence of KIEs in a thermophilic ADH from *Bacillus stearothermophilus* (bsADH) [36]. These experiments found that within this enzyme’s physiological temperature range (30–65 °C), the 1° KIEs were nearly independent of temperature and the mSSEs were inflated. Below 30 °C, however, the 1° KIEs showed a temperature dependence and the mSSEs were within error of the semi-classical value. The temperature independent 1° KIEs, along with the inflated mSSEs, in the physiological temperature range suggested that in that range, the enzyme adopted a conformation that was well-suited for tunneling, but below the physiological temperature range, a sort of phase transition left the enzyme in a conformation that was not suitable for tunneling.

Since that time, the temperature dependence of 1° KIEs has become an important tool for understanding how enzymes modulate the reaction barrier for tunneling (see below the example of TSase), but the interpretation of experimental results—both the temperature dependence of KIEs and the inflated mSSEs—has changed with the growing acceptance of Marcus-like models for H-transfers. These models assume that tunneling is a major contributor to the reactive flux for all isotopes and in all temperature ranges accessible to enzymes. As discussed above, Marcus-like models provide a mechanism for a reaction to exhibit both temperature independent and temperature dependent KIEs, depending on the population distribution along the DAD coordinate. We recently applied a numerical fitting of the Marcus-like model [54] to the 1° KIE data from wt bsADH, as well as those from a series of mutants [58]. The fittings showed that the wt in its physiological temperature range had a very narrow distribution along the DAD coordinate (at a relatively short DAD) and that this distribution did not change significantly with temperature, thus leading to temperature independent KIEs. At low temperatures, though, the enzyme adopts a conformation such that the distribution along the DAD coordinate is greatly perturbed and the system appears to undergo reaction by two distinct pathways. One lower energy population tunnels inefficiently from a long DAD, while the other reacts by heavy atom motion shortening the DAD so much that the hydride is practically over the barrier. The population with the short DAD is somewhat higher in energy, so increasing temperature increases the population at that DAD, leading to smaller KIEs at higher temperatures. We found qualitatively similar results when applying the fitting procedure to a series of active site mutants, although the mutations tended to flip the temperature ranges where KIEs were temperature independent with those where they were temperature dependent.

One of the most surprising and counterintuitive conclusions based on this model was that at high temperatures, reactions with temperature dependent KIEs must occur from a shorter DAD than similar reactions with temperature independent KIEs. Within the mathematical form of Marcus-like models (Equation (21)), the only way to achieve a small KIE is to achieve a short DAD. Thus, if at a given temperature some reaction (reaction A) has a smaller KIE than another reaction (reaction B), one can conclude that on average, reaction A occurs from a shorter DAD than reaction B at that temperature. This was a most surprising result because wt enzymes often exhibit temperature independent KIEs under physiological conditions, while active site mutants like those in the bsADH [58] and other systems [69,70] often show temperature dependent KIEs. Intuitive arguments had suggested that the role of active site hydrophobic residues was to maintain a short DAD and that mutations that made those residues smaller lengthened the DAD, leading to the observed temperature dependence of the KIEs. One study [56], however, found direct evidence that decreasing the size of a hydrophobic active site residue (V106A morphinone reductase) *decreased* the DAD. Based on these findings, one can rationalize that, given the many complex interactions in an enzyme active site, decreasing the size of a side chain may not have the direct effects on DAD that intuition would predict. 

Another important result of Marcus-like models is that D and T must tunnel from shorter DADs than H. Knowing this, and reassessing the many mSSE data from ADHs, Klinman proposed an explanation for the inflated mSSEs that modified the traditional model of tunneling and 1°–2° coupled motion [57]. The H/T KIEs with H-transfer from all the different studies had relatively constant magnitude, near the EIE (1.35), but the D/T KIEs with D-transfer were all diminished from their expected values; the diminished D/T values, therefore, appeared to cause the varying levels of inflation in the mSSEs. The new proposal suggested that the D at the 1° position in those D/T measurements has to tunnel from a shorter DAD and the requirement of a shorter DAD sterically hindered the rehybridization of the donor carbon, thus lowering the KIE. We recently tested this notion in the reverse reaction (benzaldehyde to benzyl alcohol) and found that indeed, the 2° H/T KIE is smaller when D is the transferred atom. Furthermore, we developed a computational model, based on the ideas of Marcus-like models and a shorter DAD for D-transfer, which explained the many different seemingly-incoherent data for yADH. In a relatively simple and intuitive model, we reproduced the 2° KIEs, the inflated mSSEs, and even the Hammett substituent effects, thus bringing together decades of confusing data on this enzyme into a single model [71].

One of the most important open questions regarding ADH is why it is so unique in its display of inflated mSSEs. A number of systems, both enzymatic [72,73] and non-enzymatic [28], display 2° KIEs outside of the range of the EIE to unity, but very few other systems [74] have exhibited inflated mSSEs, despite the ubiquity of tunneling. Another important area for exploration is the question of how conditions and mutations that enzymologists intuitively expect to lead to longer DADs seem to create situations where the DAD reaches smaller values [54,56]. Is this an artifact of a model that is poorly suited for the experimental data? Or is this a real physical phenomenon that should be investigated further? Both experimental and theoretical avenues of exploration are likely necessary to answer these problems.

## 4. Thymidylate Synthase

Thymidylate synthase (TSase) catalyzes the reductive methylation of 2′-deoxyuridine-5′-mono­phosphate (dUMP) using N^5^,N^10^-methylene-5,6,7,8-tetrahydrofolate (CH_2_H_4_folate) as both methylene donor and hydride reductant, thus forming 2′-deoxythymidine-5′-mono­phosphate (dTMP) [75]. This reaction constitutes the last committed step of *de novo* biosynthesis of one of the building blocks of DNA; thus its activity is crucial to most living organisms. TSase is a common target of chemotherapeutic drugs since it is overexpressed in actively proliferating cells [76].

**Scheme 2:**
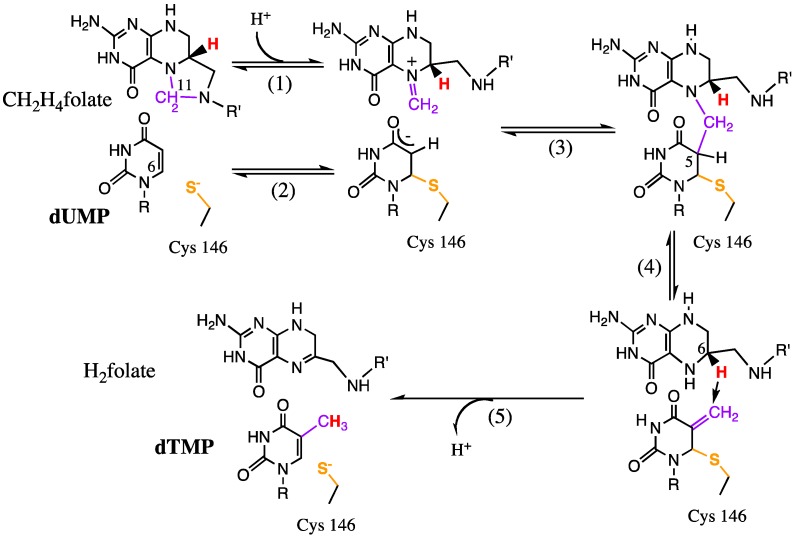
The proposed chemical mechanism of *E. coli* TSase.Reproduced with permission from ref. [8].

The TSase-catalyzed reaction involves a series of bond cleavages and formations (Scheme 2) including two different C-H bond activations: a reversible proton transfer (step 4 in Scheme 2) and an irreversible, rate-limiting hydride transfer (step 7 in Scheme 2). A number of excellent studies have probed the overall chemical mechanism of this reaction using steady-state and pre-steady-state kinetics, α-2° hydrogen KIEs and crystallographic studies [77,78,79], but here we will focus on the use of isotope effects to explore the physical details of the two C-H bond activations by both experimental and computational methods.

### 4.1. Hydride and Proton Transfers

**Figure 5:**
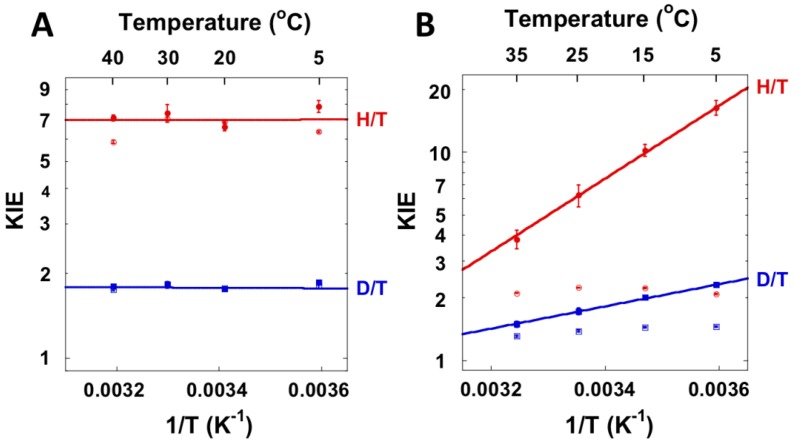
1° KIEs on the hydride transfer (**A**) and the proton transfer (**B**) catalyzed by wt *E. Coli* TSase. The empty and filled points are the observed and intrinsic KIEs, respectively. Reproduced with permission from ref. [83].

Spencer *et al.* [80] measured KIEs on the hydride transfer (Step 5 in Scheme 2) in *E. coli* TSase and found that the KIEs on *k*_cat_ and *k*_cat_/*K*_M_ were equivalent and relatively large, suggesting that the hydride-transfer step is rate-limiting for both rate constants and the observed KIEs are close to the intrinsic value (*i.e.*, ^D^*k*_cat_ = ^D^(V/K) = *k*_H_/*k*_D_). We measured KIEs on the hydride transfer over a temperature range of 5–40 °C and used the Northrop method [Equation (18)] to extract intrinsic KIEs [81]. The observed KIEs were equal to the intrinsic KIEs at the physiological temperatures (20–30 °C), substantiating the findings of Spencer *et al.* (Figure 5). However, at elevated or reduced temperatures minor commitments appear, partially masking intrinsic KIEs. While the initial velocities over a range of temperatures showed that the reaction required a small activation energy (about 4.0 kcal/mol), the intrinsic KIEs were temperature independent, indicating that the hydride tunnels from a narrow distribution of DADs that is unaffected by temperature. 

Unlike the hydride transfer, the proton transfer (step 4 in Scheme 2) is reversible, has complex commitments, and is not rate-limiting, making it difficult to measure KIEs on this step. Initial studies with saturating CH_2_H_4_ folate, for example, found 1° KIEs to be unity because of large commitment [78]. Our studies showed that the observed KIE on the proton transfer varies with the concentration of CH_2_H_4_ folate due to the sequential binding order of dUMP and CH_2_H_4_ folate [82]. Thus, we were able to measure 1° KIEs on the proton transfer using a minimal concentration of CH_2_H_4_ folate, which allowed the reaction to proceed but diminished the commitment factors [83]. The Northrop method was still valid as a means to extract intrinsic KIEs for this step because we had previously found the EIE to be unity [82]. Interestingly, intrinsic KIEs on this step were steeply temperature-dependent, contrary to the hydride transfer step, which has temperature independent KIEs (Figure 5). Numerical fitting of these KIEs to a Marcus-like Model suggested that the proton transfer occurs from at least two populations with different DADs, unlike the single narrow population for the hydride transfer [54]. These observations show that the temperature dependence of the KIEs do not simply reflect the overall flexibility of the enzyme, but reflect the means by which the enzyme catalyzes a specific step in a reaction. In this case, the enzyme employs different strategies to activate two different C-H bonds, with the active site more rigid for the rate-limiting hydride transfer.

### 4.2. Mutagenesis Studies

**Scheme 3:**
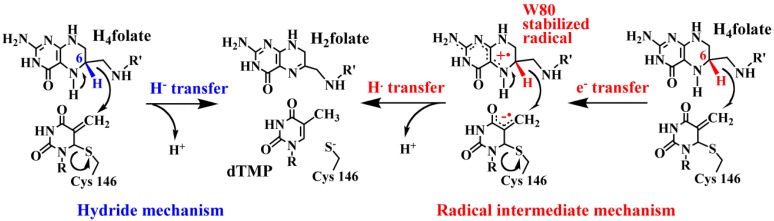
Two proposed mechanisms for the reduction of the exocyclic methylene. Reproduced with permission from ref. [8].

KIEs and their temperature dependence are excellent reporters on the nature of bond activation since they are extremely sensitive to the reaction’s potential surface. KIEs thus allow one to observe the effect of mutations on the nature of H-transfer (e.g., the hydride and proton transfers in TSase). For example, two distinct mechanisms (Scheme 3) had been proposed for the reduction of the exocyclic methylene in Step 5 of Scheme 2: a two-step radical mechanism where W80 stabilizes the radical intermediate of H_4_ folate [84] and a one-step hydride transfer where the role of W80 is to orient L143, which protects the exocyclic methylene intermediate from being attacked by a nucleophile [85]. In order to distinguish the two proposed mechanisms, we mutated the residue W80 to methionine (W80M), which cannot stabilize the radical intermediate [86]. Therefore, if the W80 is, indeed, necessary to stabilize the radical, W80M would show significant differences in KIEs and their temperature dependence because of the radical’s instability. We found that the intrinsic KIEs of the mutant were identical to the wt between 5–40 °C, suggesting that the mechanism of reduction was unaffected by the mutation, lending support to the one-step hydride transfer mechanism.

**Figure 6:**
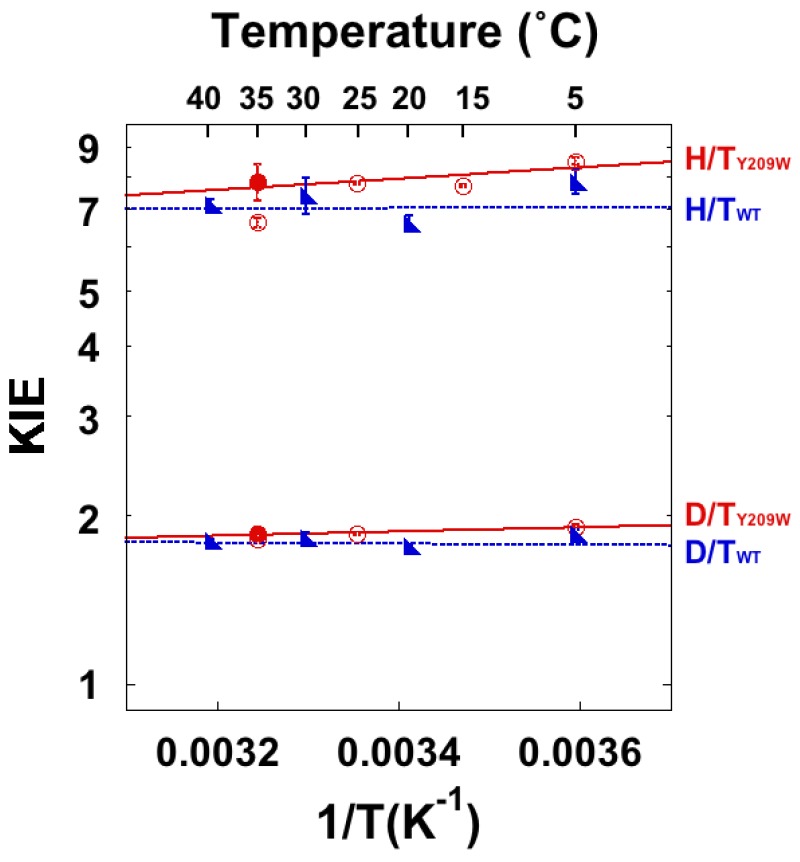
Observed (empty circles) and intrinsic (filled circles) KIEs on the hydride transfers in the wt (blue triangles) and Y209W (red circles). Reproduced with permission from ref. [87].

In another example, the KIEs of mutants illuminated the role of protein motions throughout the enzyme in the hydride transfer step. In that study, we mutated a residue distant from where the hydride transfer takes place (Y209W) [87]. A crystal structure of the mutant [88] was remarkably similar to the wt, indicating no significant conformational change. Analysis of the anisotropic B-factors, on the other hand, indicated a reduction in rigidity throughout the enzyme. The observed disruptions to the correlated vibrations of several protein segments allow thiols in solution to compete for the exocyclic methylene intermediate prior to the hydride transfer (step 5, Scheme 2). Thus, the mutation disrupts the enzyme’s dynamics 8 Å away, hindering the enzyme’s ability to protect the intermediate from competition with external nucleophiles. The minor changes in intrinsic KIEs (Figure 6) for this mutant indicated only minor effects on the hydride transfer *per se*. Since the intrinsic KIEs and their temperature dependence are expected to be affected by fast dynamics (femto-nano-seconds), it appears like those fast motions that directly affect the fast bond cleavage are not altered in the active site. However, the mutation caused a 500-fold decrease in *k*_cat_ and the observed KIEs were dramatically altered, indicating effects on protein dynamics at slower time scales. The mutant exhibits a higher enthalpy of activation for *k*_cat_ than the wt. Thus, one can conclude that the mutation hinders the enzyme’s ability to reach the TRS but has only modest effects on the DAD distribution at the TRS. The increased disorder evident in the crystal structure, then, appears to correspond primarily with the relatively slow dynamics that affect macroscopic rate constants, but does have a slight effect on the fast dynamics associated with the H-transfer. The mechanism by which this remote mutation affects the DAD distribution is a curiosity that provides a very important avenue for future research. The phenomenon of remote mutations having long range effects occurs in other enzymes, such as DHFR [89], SBL-1 [37,90], and others. The temperature dependence of intrinsic KIEs serves as an important tool in studying these and many other enzymes.

### 4.3. High-Level Simulations

To further explore the physical mechanism of the hydride transfer in TSase, we conducted hybrid quantum mechanics/molecular mechanics (QM/MM) calculations [46,91,92]. The QM/MM calculations suggested that the hydride-transfer occurs by a concerted 1,3-S_N_2reaction,wherethe hydride transfer is coupled to C6-S bond cleavage (Step 5, Scheme 2). The calculations also identified several active-site residues, especially R166, that polarize the C6-S bond and stabilize the resulting sulfur anion, thereby reducing the free energy barrier [92]. We also calculated KIEs on the hydride transfer in the experimental temperature range using ensemble-averaged variational TST with multidimensional tunneling [46]. Our calculations reproduced the experimentally observed temperature independent KIEs (though not their magnitude) and further suggested that the hydride transfer is dominated by trajectories that occur by quantum mechanical tunneling, with around 91% and 80% tunneling for protium and tritium, respectively. Thermal fluctuations in the active site cause marginal changes in the DADs, thus enabling tunneling by all isotopes of hydrogen at all temperatures. These calculations identified some key protein promoting vibrations, such as movements of R166 that polarize the C6-S bond. The calculation was repeated at different temperatures, and suggested that the potential of mean force (PMF) for this step does not vary with temperature. A possible interpretation of the invariant PMF is that the enzyme “thermostats” the vibrational motions in the active site, which has a “buffering effect” on the vibrations affecting the hydride transfer, leaving them unchanged at different temperatures. This suggestion is different than that proposed by similar calculations of DHFR [45], and more studies are needed to examine how general the sources of temperature independent KIEs are. Recently, additional QM/MM simulations opened new directions for studies of the proton transfer step [93] and experimental studies are underway to examine the role of R166 in stabilizing the C146 anion proposed to form at different points during the reaction.

## 5. Conclusions

KIEs provide unique data on the chemical step in enzyme catalyzed reactions. Two examples were brought above to demonstrate some of the uses of KIEs (*i.e.*, ADH and TSase). Studies of ADH demonstrated how 2° KIEs can assess TS structure and how to interpret 2° KIEs within the framework of different models. The studies of TSase demonstrated the use of temperature dependence of 1° KIEs in the investigation of both the hydride and proton transfers involved in that reaction. Combining experimental KIE measurements with QM/MM simulations has clarified the molecular mechanism, possible role of dynamics, and transition state structures for the enzyme, paving the way for the production of new transition state analog inhibitors with possible medical value.

While KIE studies have helped tremendously to understand enzyme mechanisms, important questions remain over the interpretation of the temperature dependence of KIEs. Marcus-like models seems to provide a framework for modeling enzymatic hydrogen transfers, and so far appear to be able to fit all published experimental findings (without invoking non-statistical dynamics coupled to the H-transfer). However quantitative attempts to fit experimental data to such models have yielded surprising results that some perceive as counter-intuitive [37,54]. Additional work is needed to test those models and find a comprehensive model with broad applications. Additional guidance from high level calculations may be particularly helpful in this regard. Some simulations [47], for example, have used similar ideas to Marcus-like models to explain the varied temperature dependence of KIEs, but other computational studies [45,46,49] have relied on different approaches to reproduce the experimental data. A theory that best explains the experimental results might eventually be different for different enzymes, or represent a unified model for temperature dependence of KIEs in all systems (enzymatic and non-enzymatic). The experimental community will benefit from better understanding of what physical phenomenon the temperature dependence of KIEs is measuring. Determining how different enzyme environments give rise to different KIEs may assist rational drug design to take advantage of the unique mechanisms of enzyme catalysis, and for biomimetic catalysts to bridge the present gap in catalytic efficiency versus natural catalysts [94,95].
